# Minimal Camera Networks for 3D Image Based Modeling of Cultural Heritage Objects

**DOI:** 10.3390/s140405785

**Published:** 2014-03-25

**Authors:** Bashar Alsadik, Markus Gerke, George Vosselman, Afrah Daham, Luma Jasim

**Affiliations:** 1 Department of Earth Observation Science, Faculty ITC, University of Twente, 7500 AE Enschede, The Netherlands; E-Mails: m.gerke@utwente.nl (M.G.); george.vosselman@utwente.nl (G.V.); 2 Department of Surveying, College of Engineering, University of Baghdad, Baghdad 10071, Iraq; E-Mails: afrah.daham@gmail.com (A.D.); j.lumakhalid@yahoo.com (L.J.)

**Keywords:** camera network, visibility, ellipsoid of error, point cloud, FIS

## Abstract

3D modeling of cultural heritage objects like artifacts, statues and buildings is nowadays an important tool for virtual museums, preservation and restoration. In this paper, we introduce a method to automatically design a minimal imaging network for the 3D modeling of cultural heritage objects. This becomes important for reducing the image capture time and processing when documenting large and complex sites. Moreover, such a minimal camera network design is desirable for imaging non-digitally documented artifacts in museums and other archeological sites to avoid disturbing the visitors for a long time and/or moving delicate precious objects to complete the documentation task. The developed method is tested on the Iraqi famous statue “Lamassu”. Lamassu is a human-headed winged bull of over 4.25 m in height from the era of Ashurnasirpal II (883–859 BC). Close-range photogrammetry is used for the 3D modeling task where a dense ordered imaging network of 45 high resolution images were captured around Lamassu with an object sample distance of 1 mm. These images constitute a dense network and the aim of our study was to apply our method to reduce the number of images for the 3D modeling and at the same time preserve pre-defined point accuracy. Temporary control points were fixed evenly on the body of Lamassu and measured by using a total station for the external validation and scaling purpose. Two network filtering methods are implemented and three different software packages are used to investigate the efficiency of the image orientation and modeling of the statue in the filtered (reduced) image networks. Internal and external validation results prove that minimal image networks can provide highly accurate records and efficiency in terms of visualization, completeness, processing time (>60% reduction) and the final accuracy of 1 mm.

## 1. Introduction

The documentation of archeological elements through advanced imaging techniques, finally leading to a detailed 3D model of the objects of interest, is currently a hot topic both in commercial as well as in scientific communities. Some examples of using the 3D technology in this field are listed in [1] and there is even a conference series devoted to this topic [2]. Such a 3D representation is used for visualization, archaeological documentation, restoration, or preservation purposes. In general we use the term *image based modeling* [3,4] if we refer to the entire workflow from image acquisition, to image calibration and orientation, to image matching and meshing, or to CAD-like object reconstruction and parameterization.

Although software tools which offer support for the entire workflow are available nowadays, a good planning of the initial image acquisition is still necessary in many applications such as in archeology in order to achieve the desired accuracy and reliability of subsequent image processing steps. Moreover, portable physical cultural finds need to be documented first *in-situ* locations before preserving them in heritage collections and museums [5]. This mission of field data capture in excavation sites is preferred to be automated in order to save time during the capture and ensure adequate data [6]. The mentioned photo acquisition planning demands experience and knowledge in the field of photogrammetry.

In the literature few recently presented papers deal with the proper selection and acquisition of images among a large dataset for 3D image-based modeling. Hosseininaveh, *et al*. [7] introduced a method called Image Network Designer (IND) for the modeling of a museum artifact. That paper lacks a deep investigation on the accuracy indices after the network reduction and the experiment was tested on small size artifact with only a few images. Wenzel, *et al*. [8] presented a guideline for image data acquisition called “one panorama each step”. They discussed extensively how to find a compromise between large and short base imaging configurations.

Previously, we introduced the minimal camera network technique [9] by reducing a pre-designed (simulated) dense imaging network. The redundancy in the information from the dense network will provide freedom for selecting the suitable and sufficient images for the subsequent 3D modeling. This technique is useful, but it fails to accommodate better intersection geometry of rays between cameras and object points and this can result in gaps in the final 3D models. In practical projects it is normally not necessary to reduce the number of images if those have already been acquired. Our aim is to support the image acquisition phase from the beginning by providing a method which supports the photographer to take the most suitable images, and at the same time to reduce the total number of images to a minimum. This fast, reliable acquisition of images with the minimum number of images is important for the digital documentation of archeological or heritage objects. To this end we propose a two-step approach where first a video of the object of interest is acquired, followed by a fully automatic image network planning phase (see [9] for details). In this paper, we present two advanced methods of filtering a dense camera network to a minimal set where a complete 3D model with even strict accuracy demands can be acquired. These methods will enhance the approach introduced in [9] which is based on only satisfying the coverage requirements. The first proposed method is based on satisfying the accuracy indices in the object points while the second method is based on finding a compromise between the coverage and accuracy by a fuzzy inference system (FIS). The FIS use rules combining the requirements of the uncertainty in the viewing cameras, the number of points per image and their distribution as will be discussed in the following Section 2. A case study of cultural heritage object will then be tested to verify the new proposed techniques.

## 2. Methodology

In order to find the minimal camera network for the 3D modeling of cultural heritage objects, a dense imaging network is filtered on the basis of removing redundant cameras in terms of coverage efficiency and the impact on the total accuracy in the object space or the uncertainty of cameras orientation. The following sections describe the methodology for computing the visibility status and the camera reduction (filtering) technique.

### 2.1. Visibility Requirement

**Figure 1.**
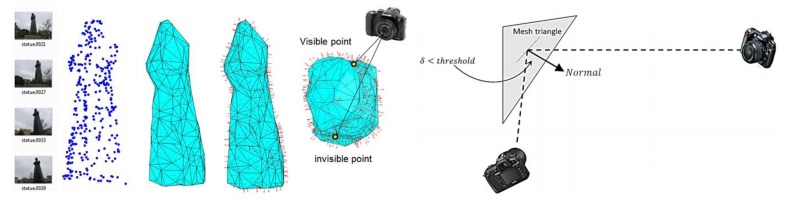
Visibility by using the triangular surface normal vectors.

The visibility of object points from the different camera locations is an important factor during the design and filtering of the imaging network. In other words, we should carefully compute for every part of the object of interest, the imaging cameras according to their designed orientation. Different methods can be used to test the visible points like the HPR method [10] or the surface-based method which is used in this paper. First a triangulation surface is to be created and the normal vector for each triangular face is computed. These normal vectors are used to test the visibility of points in each camera as shown in Figure 1 for a statue example [11]. The decision of considering points as visible or invisible depends on the absolute difference between the orientation of the camera optical axis and the face normal direction N_dir_. This difference is compared to a threshold (like <90°) to decide the visibility status. It must be noted that the threshold angle is also related to the baseline/depth (B/D) ratio and its magnitude can be selected to satisfy the desired small ratio for the 3D modeling [12]. Finally, this method is expected to avoid the self-occluded parts of the study objects since the visibility is based on the geometry of the object surface as shown in Figure 1.

### 2.2. Minimal Camera Network and Filtering

The aim of this research is to introduce a new method of finding the minimum set of cameras within a pre-designed dense imaging network, which guarantees the sufficient coverage and accuracy of the 3D modeling of heritage objects. Fraser [13] stated that high accuracy can be achieved with a large B/D ratio. However, it is not useful if the ultimate task is to derive a highly detailed 3D model by the dense matching techniques: that would require a short base imaging network [2,12,14].

Previously, we published our filtering method for a dense imaging network [9,11]. The method was based on filtering out the redundant cameras with the least imaging points (filtering for coverage). In this paper two new strategies of filtering will be presented, the first strategy is to filter out the redundant cameras with the least impact on the point cloud accuracy (*σ_x_*, *σ_y_*, *σ_z_*). The second strategy is to use a rule based method of fuzzy logic [15] to assign the suitability of each camera in the sense of uncertainty, number of imaged points and their distribution. Both methods have been run iteratively because the number of cameras viewing the same point will be changed every time a camera is filtered out.

#### 2.2.1. Filtering Based on the Accuracy of Object Points

**Figure 2.**
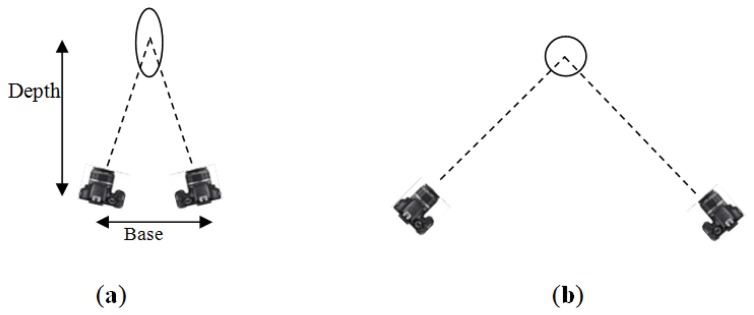
Intersection geometry and error plot. (**a**) Weak intersection with small base\depth ratio; (**b**) Strong intersection with large base\depth ratio.

The motivation of using the filtering for point accuracy is based on the well-known relation between the ray intersection geometry and accuracy at the intersection point as shown in Figure 2. Therefore, the technique prefers to cancel the cameras of the weak intersection geometry while preserves the desired B/D ratio.

**Figure 3.**
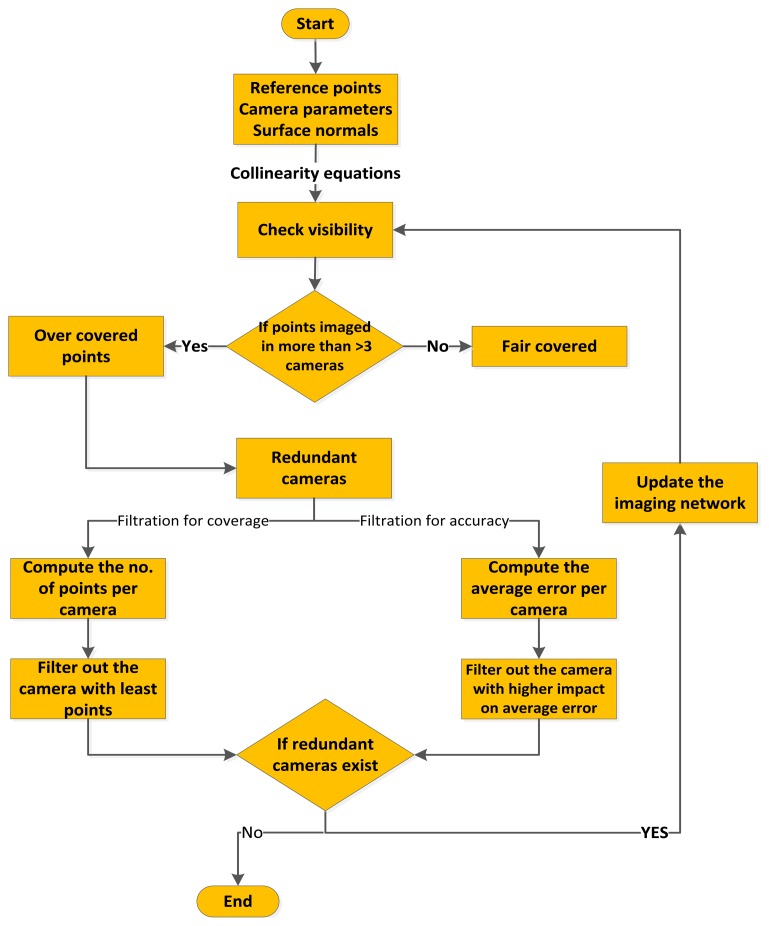
A methodology flowchart of filtering for coverage and accuracy requirements.

Accordingly, the filtering is based on evaluating the total error in the object space and computing the effect of each camera on this error. The least effective redundant camera in terms of accuracy will be neglected. The whole procedure of filtering will be iterated until reaching the desired accuracy (by error propagation) or when no more redundant cameras are found in the imaging network. The algorithm implementing the old strategy based on coverage and the new strategy based on accuracy is illustrated in Figure 3.

The summarized procedure is:

-Prepare the input information of point cloud, designed external and internal camera parameters, and the initial surface normals.
-Project the points back to the images by collinearity equations [16] to decide the visible and invisible points per camera.
-Classify the points as over-covered points if they imaged by more than three cameras [17]. Otherwise, they are classified as fair-covered points. Actually, during the initial block setup, it was assured that there are at least three rays per point and within this procedure we also make sure that any point is visible in three images as well.
-Consequently, the cameras involved in imaging over-covered points are classified as redundant camera and is subject to filtering out according to the accuracy requirements.
-To filter the redundant cameras based on accuracy, the covariance matrix of every point per camera is computed [18]. The worst error is evaluated and assigned to the redundant camera. The redundant camera with the least impact on the total accuracy will be filtered out. This impact is a measure of the accuracy before and after the reduction. The filtering is iterated until no more redundant cameras exist.

Preferably, additional connection cameras to be added when modeling objects of steep connected faces like buildings [9].

#### 2.2.2. Filtering with Fuzzy Logic Inference

The motivation of using this method is to find a compromise between the demands of good coverage and high accuracy in one filtering model. Sivanandam, Deepa and Sumathi [15] stated that fuzzy logic provides an inference method that enables proper human reasoning abilities. However, building a fuzzy inference needs to design a membership function for the inputs (linguistic variables). These variables are labeled like (High accuracy, Low accuracy) where their effect mathematically defines the fuzzy domain. Hence, the humanly explanation of a variable can be translated into a mathematical language.

The developed FIS will use specific fuzzy rules to produce the suitability measure (between 0 and 1) of each camera. These rules are set after testing several types and the user can modify the logical rules relying on the type of the problem. The proposed FIS filtering includes a Mamdani-type [19], four input parameters and one output module.

**Figure 4.**
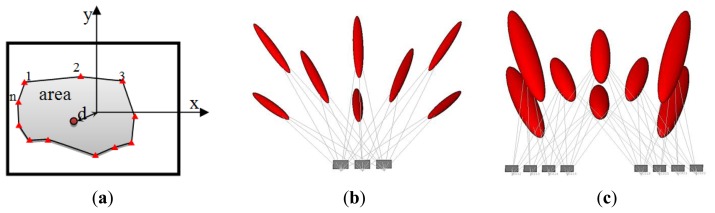
Estimated uncertainty in the object space with ellipsoid of errors. (**a**) Sketch of the input measures in image plane; (**b**) Overall vector of precision is 5.2 mm; (**c**) Overall vector of precision is 6.3 mm.

The input parameters are to be computed every iteration: the uncertainty of camera orientation, the number of visible points in each image, the proximity (d) of the image center (principal point p.p.) to the cluster of the object points in the image plane and the area covered through the distribution of the points as shown in Figure 4a.

The motive for considering these parameters is illustrated in Figure 4 where the three cameras of Figure 4b are viewing simultaneously all the eight object points, the area spanned by these points in the image planes are high and the center of the image points is close to the image p.p. The parameter of proximity can express the uniformity of the image points. This imaging configuration result in a relatively small error compared to the network in Figure 4c.

The imaging network of Figure 4c shows the camera placement aside from the middle. The average number of points viewed by the images is less, the coverage area is less and the proximity distance to the image p.p. is higher. The accuracy is degraded as the ellipsoids of errors indicate despite the higher number of cameras than the cameras of the first case in Figure 4b. This can address an important conclusion about the relation between the final accuracy of the object points to the number of points, number of cameras, area of coverage, and distribution pattern of points in the image planes.

Accordingly, the membership functions are designed by using our own intuition as discussed, which is also inspired by the work of [20]. These membership functions are a matter of change depending on the user, the experience and the problem to be solved. The uncertainty measure for every camera is the trace of the covariance matrix of the six orientation parameters (three angles and three translations). This is to be mathematically computed by using the collinearity equations and the membership function is designed to be triangular. The number of points which is visible in every image is also computed. The area spanned by these points is computed to measure their distribution over the images. This is can be done by computing the convex hull of the points as shown in Figure 4a. The output of the FIS in every iteration will indicate the redundant camera that can be canceled without affecting the coverage of points. The iteration is continued until no more redundant cameras exist just like the other filtering methods. The input parameters are standardized to the range [0, 1] for simplicity as follows:

The linguistic variables of the number of points (*Np*) are designed as:
  *low*: *Np* ≤ 0.3
  *medium*: 0.3 < *Np* ≤ 0.6
  *high*: *Np* > 0.6
The linguistic variables of the points distribution (*area*) are designed as:
  *low*: *area* ≤ 0.5
  *high*: *area* > 0.5
The linguistic variables of the camera uncertainty (*uncert*) are designed as:
  *high*: *uncert* ≤ 0.5
  *low*: *uncert* > 0.5
The linguistic variables for the proximity measure are designed as:
  *high*: *prox* ≤ 0.5
  *low*: *prox* > 0.5

**Figure 5.**
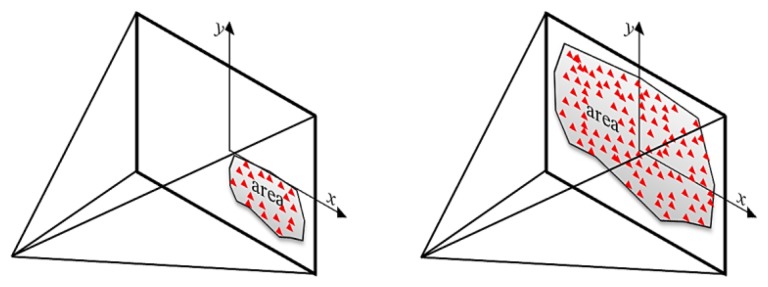
(**a**) Redundant camera; (**b**) Significant camera.

Different rules are designed to comply with the physical problem of filtering redundant cameras in a dense imaging network like the following rules (Figure 5):

(*a*) *If* (*Np is not low*) *and* (*area is not low*) *and* (*uncertainty is not low*) *then* (*filtering* = *significant camera*)
(*b*) *If* (*Np is low*) *and* (*area is low*) *and* (*uncertainty is low*) *then* (*filtering* = *redundant camera*)

To implement the inference automatically another option of using the Adaptive Neuro-Fuzzy Inference System (ANFIS) can be followed [21]. The use of ANFIS instead of using FIS will avoid the design and selection of the membership functions which might be a difficult task. With ANFIS the ranges and parameters of the membership functions are automatically computed using grid partition or clustering techniques, where a FIS implemented in the frame of adaptive network.

**Figure 6.**
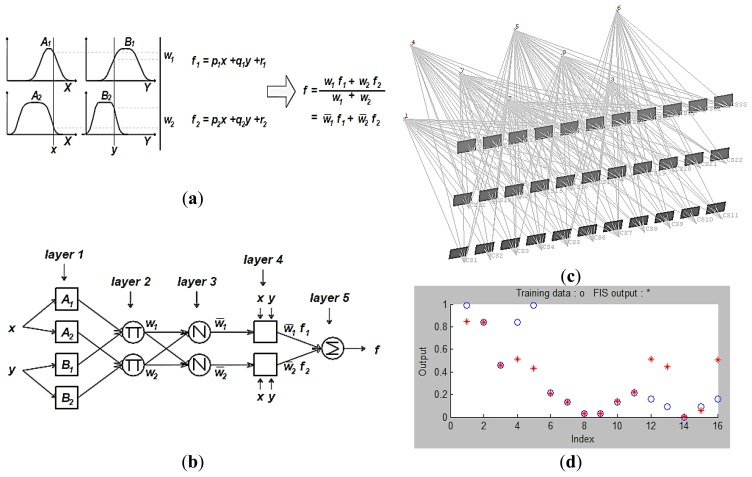
(**a**) First order Sugeno fuzzy model; (**b**) Corresponding ANFIS architecture [21]; (**c**) The data set for learning and checking of ANFIS; (**d**) The MATLAB interface for the ANFIS training and checking.

ANFIS uses a Sugeno-type fuzzy system [22], which is very suitable for tuning by optimization and they implement polynomial type output membership functions, which simplifies the defuzzification procedure. Two types of Sugeno fuzzy inference system are found (zero order with constant output and first order with polynomial output). A first order Sugeno fuzzy model is shown in Figure 6a [21,23]. The output *f* is to be derived from a given input vector [*x*, *y*]. The firing strengths *w_i_* are obtained as the product of the membership grades and the output *f* is the weighted mean of each rules output. The ANFIS network which consists of five layers is illustrated in Figure 6b. In layer 1, the membership functions are generated and the parameters involved are named premise parameters. The firing strength parameters *w_i_* are calculated in layer 2 and the ratio to the total firing strength is calculated in layer 3 for every node in the fuzzy network. The contribution of the rules to the output is to be computed in layer 4 and finally the overall output is computed in layer 5. More details about the layers of ANFIS can be found in [21,23].

**Figure 7.**
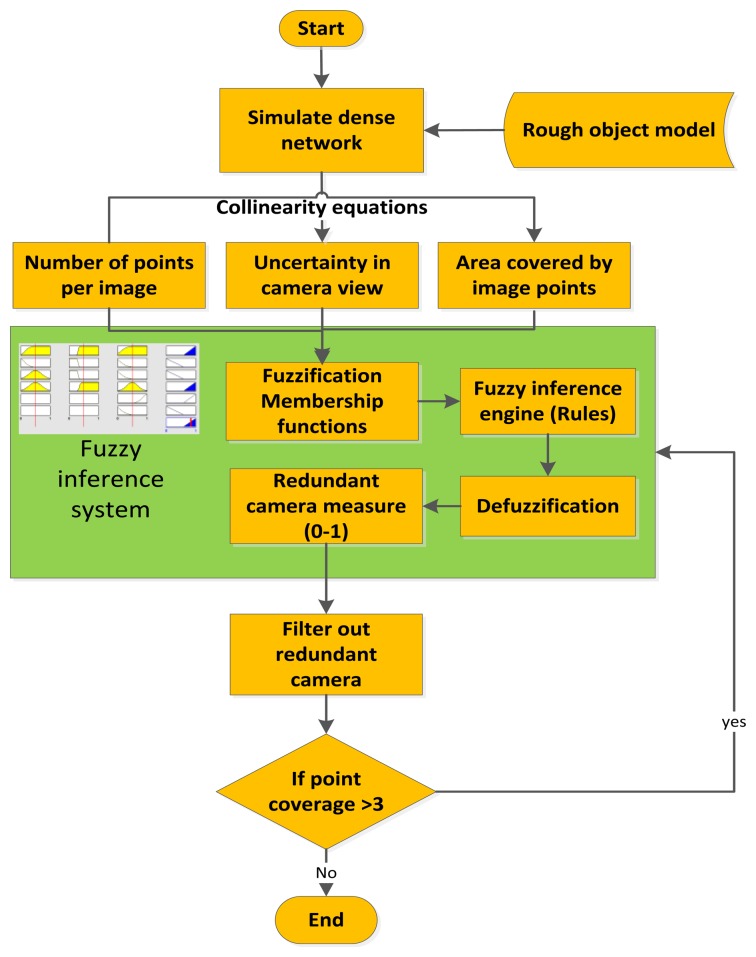
A methodology flowchart of filtering with FIS.

The first step to implement the ANFIS is the learning of information about a data set. The learning and checking for the minimal camera network is based on the simulated network of Figure 6c which consists of three strips with different angular orientations. However, more data set can also be included in the learning and checking of ANFIS which is shown in Figure 6d. The same camera filtering parameters used in the Mamdani FIS are used for the Sugeno ANFIS. The checking data is to see how well the FIS model estimates the corresponding data set and output values shows an average error of 0.22 for the model validation. The methodology of filtering by using FIS or ANFIS is illustrated in Figure 7.

#### 2.2.3. A Simulation Test

**Figure 8.**
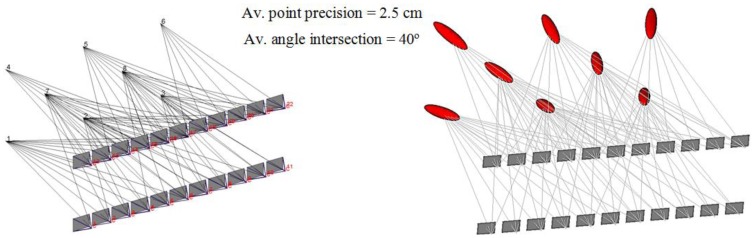
The simulated dense camera network for the verification test and the magnitude of imaging coverage and error before filtering.

To illustrate the methodology of the network reduction procedure, a simulated test is created which consists of eight points and 22 viewing cameras, as shown in Figure 8. The camera network is designed with 18 mm focal length and 22 × 14 mm^2^ format size with a B/D ratio of 80%.

To evaluate the expected error in the object points, the standard deviations are estimated by image triangulation [16,24]. Therefore, a perturbation of normally distributed errors of ±0.1 mm is added to the image coordinates. The rays in Figure 8 illustrate the number camera rays per point and their exaggerated ellipsoid of errors.

**Figure 9.**
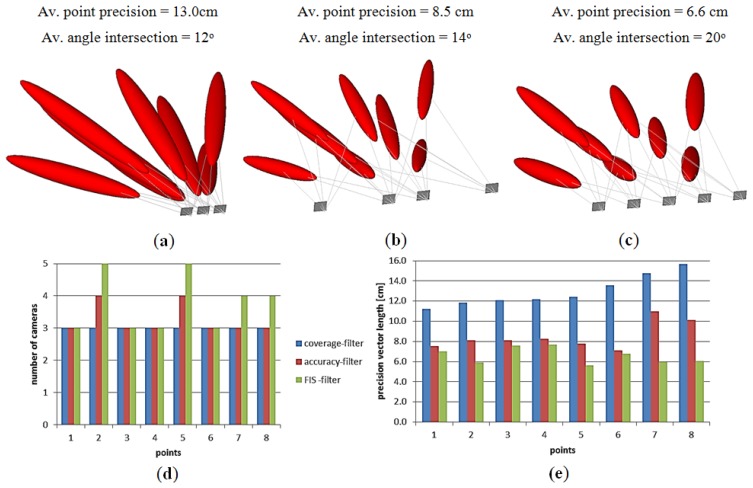
The minimal networks and the estimated error ellipsoid. (**a**) Filtered for coverage; (**b**) Filtered for accuracy; (**c**) Filtered by FIS; (**d**) The histogram of imaging cameras; (**e**) Histogram of precision.

Accordingly, the two new filtering techniques are implemented to find the minimal camera network that satisfies the coverage and accuracy requirements. The error plot ellipsoid gives a good visual aid for the comparison between the different techniques. In Figure 9a the previous technique of filtering for coverage [9] is shown where the errors are elongated and larger in the direction of the viewing rays. Figure 9b illustrates the resulting minimal network with the filtering for accuracy requirements of minimal four cameras. Figure 9c illustrates the filtered technique by using the FIS which is based on satisfying both coverage and viewing accuracy.

This simulated test showed the benefit of using each camera filtering technique in the sense of coverage and accuracy of the object points. The estimated errors seem better in the technique of filtering with FIS due to the strong ray’s intersection of 20° which means a wide base imaging. Moreover, the plots in Figure 9d,e show the dependency between the number of imaging cameras and positional accuracy beside the effect of the imaging configuration. The filtering for coverage gives a short base imaging which might be preferred for the 3D modeling purpose [8,12] despite the low accuracy. For a better understanding, a 3D modeling with minimal cameras is applied to the archeological Mesopotamian statue of Lamassu as will be discussed in the following section.

#### 2.2.4. Case Study: Cultural Heritage Statue of Lamassu

**Figure 10.**
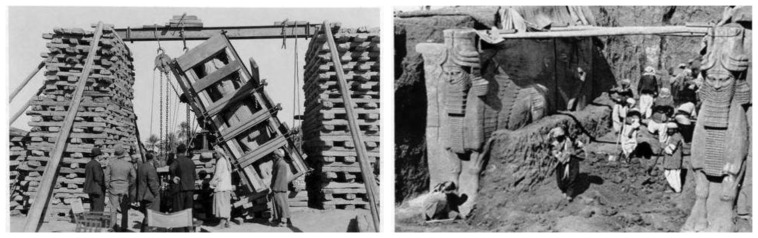
Gate of Sargon II’s citadel excavation, Khorsabad [25].

The study case of the presented approach is tested on the famous Iraqi sculpture “Lamassu” which means “protective spirit” in Akkadian. Lamassu is a human-headed winged bull of over 4.25 m in height. It dates back to the reign of Ashurnasirpal II’s (883–859 BC). In Figure 10, it can be seen how much scientific archeological benefit would to be gained if 3D digital documentation were available at the time of excavation and before moving those statues. Today, there are many pairs of these sculptures that are still in existence including those in the British Museum (London), Louvre (Paris) and Metropolitan Museum of Art (New York), as well as in the Iraqi national museum where this experiment is implemented.

**Figure 11.**
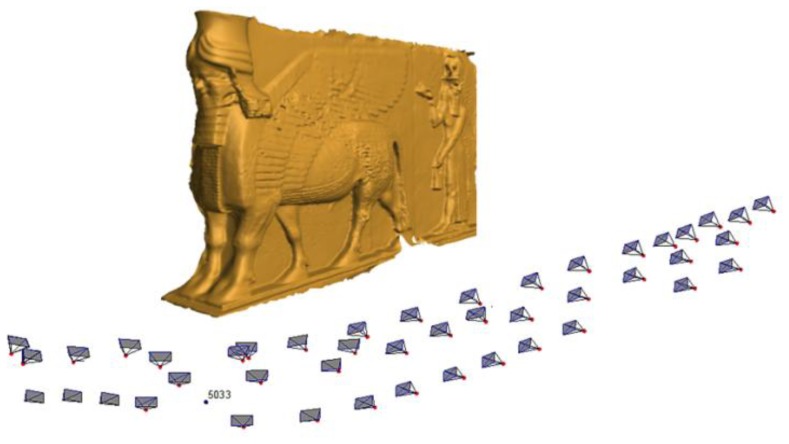
The dense imaging network configuration.

The imaging network design is planned in an ordered block that results in a ground sample distance of 1mm. The camera used is a Canon 1100D with a focal length of 18 mm which constrains the depth distance between the cameras and the statue body to 2.7 m and a scale of 1/1,500. The expected accuracy of the object points *σ_XYZ_* according to Fraser [13] is 1.7 mm as shown in Equation (1). This accuracy is complying with the recommended detailed heritage recording accuracy levels of ±2 mm according to Letellier [26]:

(1) $$\sigma_{\text{XYZ}}=(q*\text{scale}*\sigma_{\text{image}})/\sqrt{k}$$

where *q* is a design factor expressing the strength of the basic camera station configuration, *σ_image_* is the standard error in the image coordinates and *k* is the number of images per station [13]. According to our design, experience and literature, these parameter values are considered as *q* = 0.7, *σ_image_* = 1/3 pixel, and *k* = 1 respectively. The dense imaging block consists of 45 images with three strips as illustrated in Figure 11. The third strip is captured with a height of 2.5 m where a small ladder is used.

**Figure 12.**
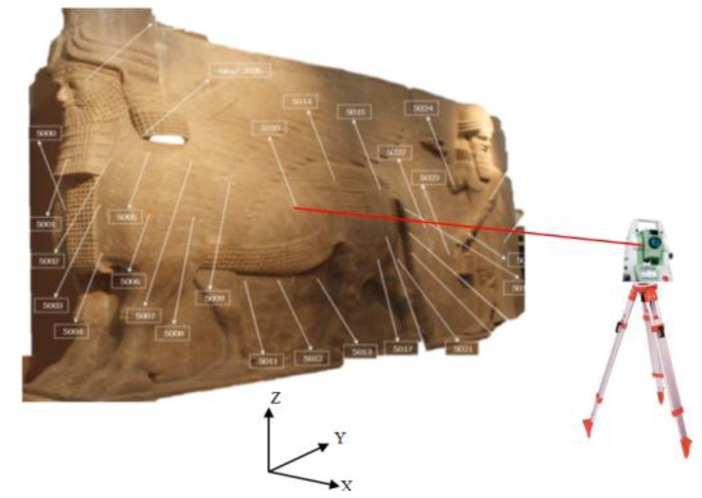
Measuring the reference points.

For the external validation and scaling, 28 reference points are fixed on the Lamassue body of paper stickers with crosses that cannot affect the physical body. A Leica TPS 400 total station was used to measure the 3D coordinates with a local datum as shown in Figure 12. The positional accuracy obtained from this survey is ±2 mm (standard deviation).

**Figure 13.**
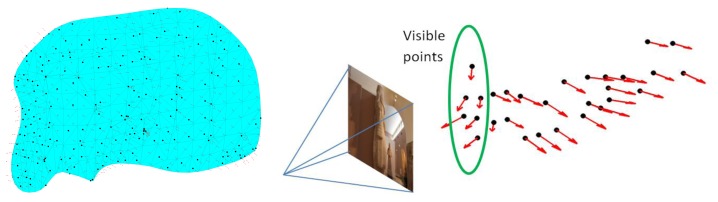
The uniform mesh of the rough model with surface normals for visibility testing.

The reference points are used to define the imaging area by a surface triangulation method using ball pivoting [27]. The reference point distribution and number is crucial for having a good coverage and to describe the rough geometry of the object. Otherwise, inadequate coverage can result in missing camera views and finally with the uncompleted 3D model. The triangular surface normals shown in Figure 13 are used to define the visibility status of the points within the imaging cameras as discussed in Section 2.1. The angular difference between the camera optical axis and the normal direction is chosen to ascertain good intersection geometry and a proper B/D ratio for the 3D modeling goal.

## 3. Results and Discussion

**Figure 14.**
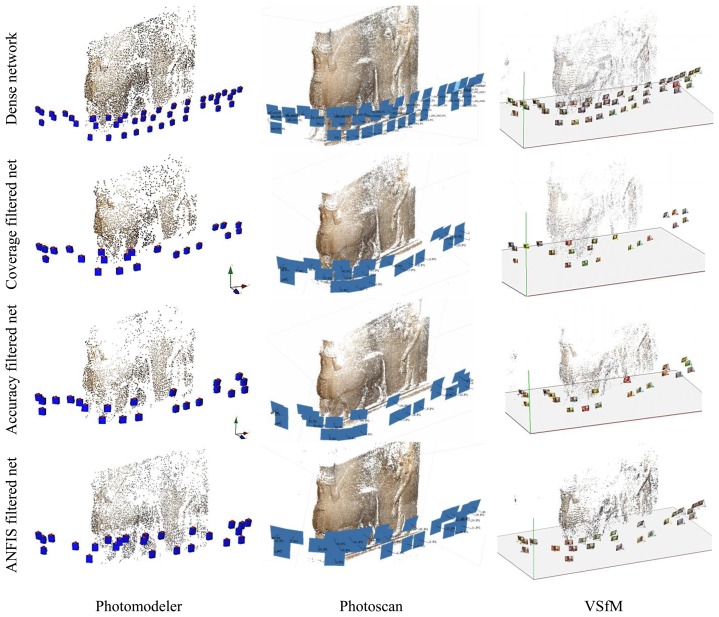
The fully automatic orientation of the four networks with different software.

The minimal networks are computed by filtering the dense network of 45 images using the three techniques and results in 22 images for filtering with accuracy condition and 25 images with the ANFIS method. To verify the efficiency of the minimal networks after filtering, we used internal and external validations. Moreover, a comparison in terms of time consuming is applied for the automatically oriented imaging networks. The orientation is done by using different commercial and open source software *Photomodeler* [28], *Agisoft* [29] and *VSfM* [30] and checkpoints are used for further accuracy evaluation. The results of the automated orientation of the three software packages are shown in Figure 14 where the whole image data sets are well oriented.

**Figure 15.**
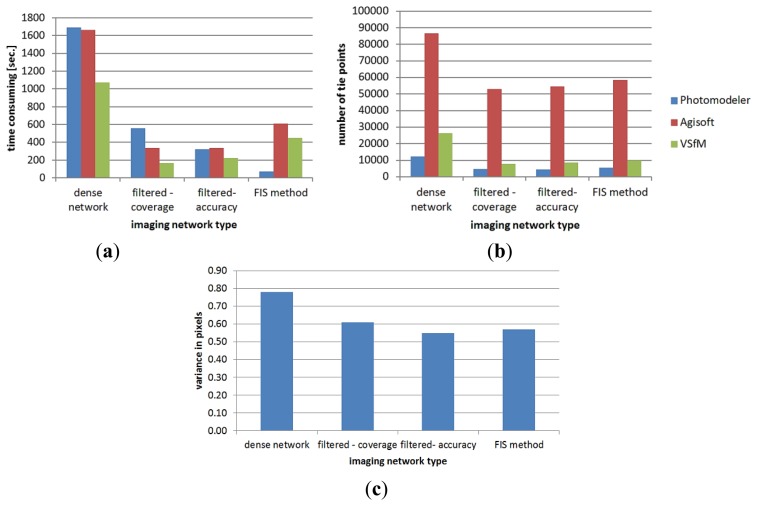
Comparison of the image orientation with different software. (**a**) Consumed time; (**b**) Number of tie points; (**c**) Reference variance after image orientation.

Figure 15 summarizes the results of the automated orientation in terms of time consuming and the number of tie points. The results show the output of the three state-of-the-art software for the three minimal imaging networks. The dense network needs more processing time as shown in Figure 15a. This is due to the time dependency between the number of images *n* and the computation cost *O*(*n*^2^) in the image correspondences matching [31]. The reference variance after the orientation of each imaging network is also shown in Figure 15c.

**Figure 16.**
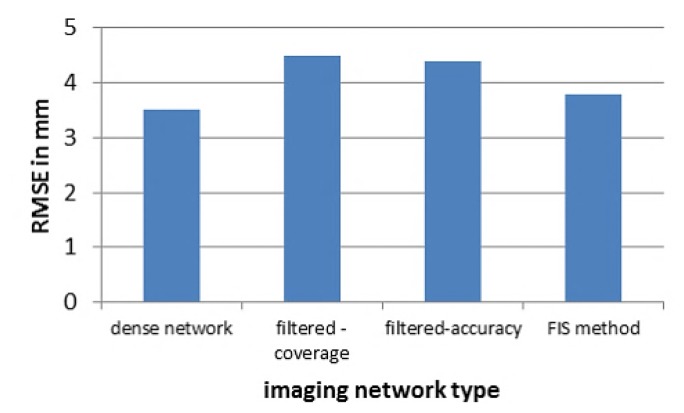
RMSE of the checkpoints.

For the external validation, the orientation is achieved in two steps. Firstly a relative orientation is done and then followed by an absolute orientation [16] by selecting 28 well distributed reference points. The results show that the filtered networks can give a close accuracy indices to the dense network despite the large reduction in the number of images (from 45 to 22). Figure 16 illustrates the root mean square error RMSE in the four networks by using 14 control points and 14 checkpoints. The average RMSE was less than 5 mm.

The relative accuracy is evaluated in the three networks as well. The longest distance between two reference points is measured from images and compared to its reference length of the total station. The computed relative error in the three networks is 1/20,000 which indicates a highly accurate close range photogrammetric measurements.

**Figure 17.**
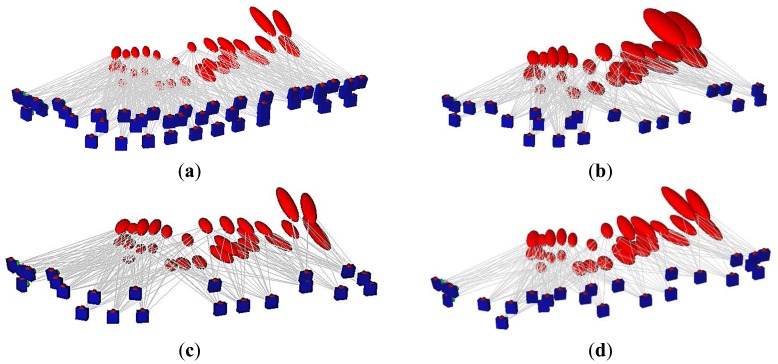
The error ellipsoids of the checkpoints. (**a**) Dense network; (**b**) Filtered network–coverage; (**c**) Filtered network–accuracy; (**d**) Filtered network with ANFIS.

The quality of all filtered networks is evaluated by computing the error ellipsoids of the reference points which resulted from the image orientation bundle adjustment as shown in Figure 17 with an exaggerated scale of 500.

**Figure 18.**
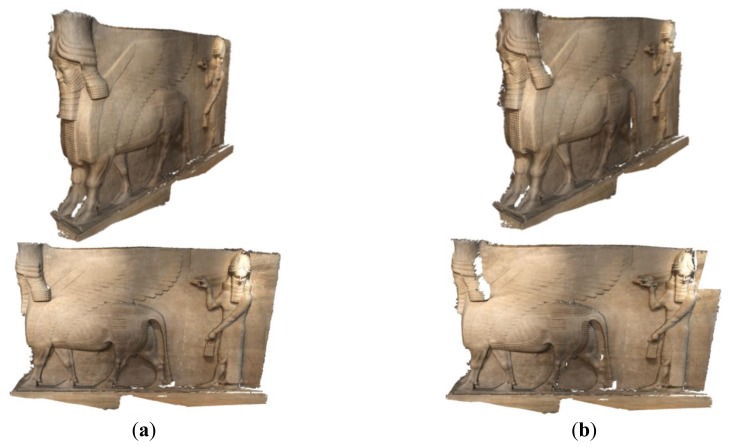

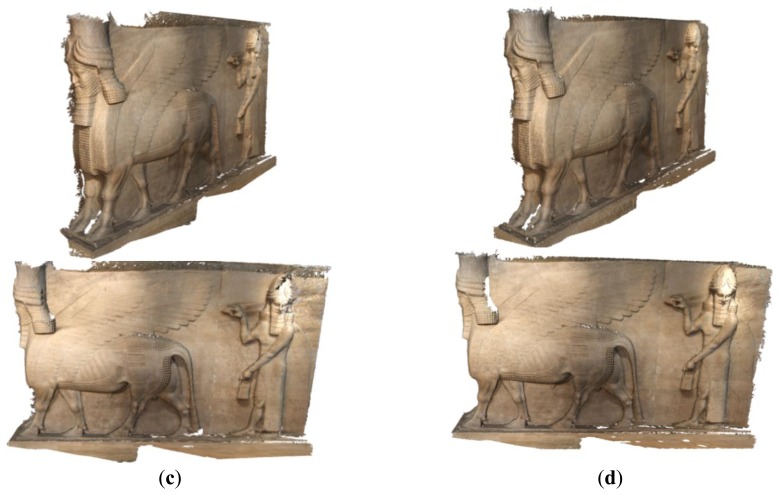
The digital 3D models in the four types of networks. (**a**) Dense network; (**b**) Filtered net-coverage; (**c**) Filtered net-accuracy; (**d**) Filtered net–FIS.

The results shown in Figure 18 of the total standard deviation in the filtered networks are about 1mm with a preference of the filtered network for accuracy. The errors are smaller than their pre-estimated values during the design step of Section 2.2.4 in Equation (1) and are still within the specification of the detailed survey of cultural heritage.

Finally, a 3D digital model is created for the final documentation of Lamassu. Figure 18 illustrates the derived 3D dense point cloud using the Agisoft photoscan software [29].

**Figure 19.**
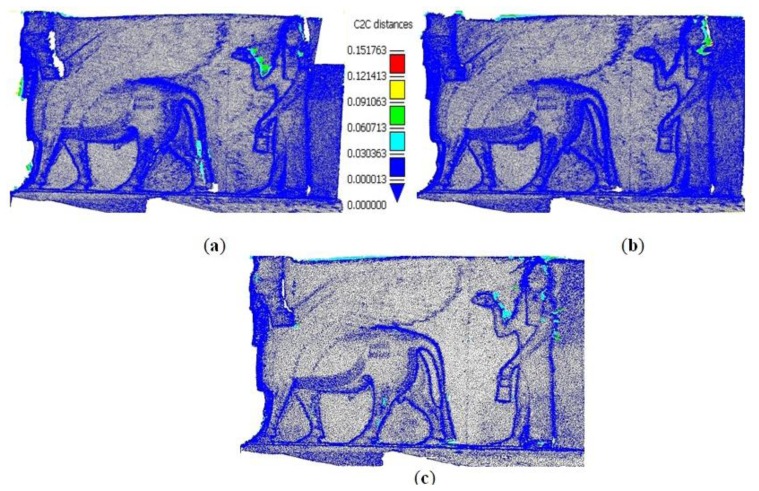
Comparison between the point clouds resulted from filtering networks and the dense network. (**a**) Filtered network for coverage; (**b**) Filtered network for accuracy; (**c**) Filtered with ANFIS.

The filtered camera network ends with a satisfactory 3D model as shown in Figure 18 compared to the full dense network. Although we do not have a ground truth for the entire surface, a comparison of the point clouds resulted from all filtered networks are compared to the resulted cloud from the full dense network. The open source software *Cloud Compare* [32] is used for this purpose. Figure 19 and Table 1 Show the point cloud comparisons in the two filtered networks where the chamfer distance is calculated by the use of octree for a speeded computation [32]. The cloud comparison of Figure 19a shows a gap near the Lamassu head and the upper right part in contrary to the new proposed techniques of Figure 19b,c.

The point cloud comparison of Figure 19 and Table 1 shows the efficiency of the produced 3D model from the filtered networks with respect to a full image data set. The large maximum distance measures are caused by the blunders resulted from the reconstruction method.

## 4. Conclusions/Outlook

In this paper, we introduced two new methods for finding the sufficient number of images for the 3D modeling of cultural heritage objects like the statues and monuments. The method is based on filtering a pre-designed dense imaging network to the minimal camera network. The minimum number of cameras was attained by using two different strategies of filtering to preserve the coverage and accuracy. The first proposed filtering method classified the object points into over-covered and fair-covered according to the minimum requirement of visibility in three images. Consequently, the cameras that contain most over-covered points are considered as redundant cameras and were investigated for filtering. The filtering is done by cancelling the redundant camera that has the least impact on the total positional accuracy as described in Section 2.2.1.

The second proposed method is developed by building a fuzzy inference system FIS or adaptive neuro-fuzzy inference System ANFIS which uses four input measures of the cameras uncertainty, the number of points per image, proximity to p.p. and their coverage area. Different fuzzy rules are composed to get the final inference of the cameras in an iterative way as described in Section 2.2.2.

The developed methods are tested on an Iraqi heritage statue of Lamassu which belongs to the Ashurnasirpal II era (883–859 BC). For analysis, we designed a dense imaging network (45 images) of three strips, captured with a Canon1100 SLR camera. Three different state-of-the-art software packages are used to automatically process the data and check the possibility of a successful image orientation with less time consuming and sub pixel variance. The filtering with accuracy requirements are tested and resulted in a reduced network of 22 images while the ANFIS method resulted in 25 images (≈50% reduction). The results show a significant decrease in the processing time (approx. 60%) which is quite promising. The final 3D models produced from the minimal imaging networks were shown in Figure18. A comparison of the 3D dense point clouds in Table 1 showed that both proposed methods are sufficient in terms of reality, visualization and completeness. Finally, the proposed filtering techniques for accuracy requirements, offered a better completed model and a higher accuracy than the other techniques as shown in Figure 19 and Table 1.

From the above discussion, we can conclude that the minimal imaging network can be computed automatically by filtering a dense imaging network which might be simulated before capture. This is proven to be sufficient for the 3D modeling purpose and accurate in terms of completeness and minimum error.

Concluding general rules to be followed for having a minimal imaging network for 3D modeling is not a practical advice. This is because of the high proficiency needed for image capturing and the effect of the object complexity on the configuration of the minimal network. Therefore, the proposed procedure of the automated simulation and filtering is more suitable to be programmed and then to be used by non-professionals in the field of cultural heritage documentation.

Further work can be investigated on the ANFIS method or other machine learning techniques to get more reliable 3D models in the sense of accuracy and object coverage. Ultimately, future work will be considered on different cultural heritage objects like ancient buildings where an additional requirements like the segmentation into facades may be applied to the task of 3D image based modeling.

## Figures and Tables

**Table 1.** The cloud to cloud distance computations.

**Imaging Networks**	**Mean Distance [m]**	**Max. Distance [m]**	**Sigma**
Dense-coverage filtered	0.002	0.150	0.009
Dense-accuracy filtered	0.001	0.130	0.008
Dense-FIS filtered	0.003	0.250	0.015
